# Fully Bayesian Inference for Structural MRI: Application to Segmentation and Statistical Analysis of T2-Hypointensities

**DOI:** 10.1371/journal.pone.0068196

**Published:** 2013-07-17

**Authors:** Paul Schmidt, Volker J. Schmid, Christian Gaser, Dorothea Buck, Susanne Bührlen, Annette Förschler, Mark Mühlau

**Affiliations:** 1 Department of Neurology, Technische Universität München, Munich, Germany; 2 TUM-Neuroimaging Center, Technische Universität München, Munich, Germany; 3 Department of Statistics, Ludwig-Maximilians-Universität München, Munich, Germany; 4 Department of Psychiatry, Jena University Hospital, Jena, Germany; 5 Department of Neurology, Jena University Hospital, Jena, Germany; 6 Department of Neuroradiology, Technische Universität München, Munich, Germany; University of East Piedmont, Italy

## Abstract

Aiming at iron-related T2-hypointensity, which is related to normal aging and neurodegenerative processes, we here present two practicable approaches, based on Bayesian inference, for preprocessing and statistical analysis of a complex set of structural MRI data. In particular, Markov Chain Monte Carlo methods were used to simulate posterior distributions. First, we rendered a segmentation algorithm that uses outlier detection based on model checking techniques within a Bayesian mixture model. Second, we rendered an analytical tool comprising a Bayesian regression model with smoothness priors (in the form of Gaussian Markov random fields) mitigating the necessity to smooth data prior to statistical analysis. For validation, we used simulated data and MRI data of 27 healthy controls (age: 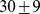; range, 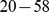). We first observed robust segmentation of both simulated T2-hypointensities and gray-matter regions known to be T2-hypointense. Second, simulated data and images of segmented T2-hypointensity were analyzed. We found not only robust identification of simulated effects but also a biologically plausible age-related increase of T2-hypointensity primarily within the dentate nucleus but also within the globus pallidus, substantia nigra, and red nucleus. Our results indicate that fully Bayesian inference can successfully be applied for preprocessing and statistical analysis of structural MRI data.

## Introduction

This work was motivated by the aim to analyze iron-related T2-hypointensity automatically in a complex set of MRI data. Increased iron content within deep gray matter (GM) regions decreases the T2-weighted MRI signal. It has been demonstrated in both normal aging and several neurodegenerative conditions [1]. Therefore, GM T2-hypointensity may be a marker of early neurodegeneration and has even been regarded of potential predictive value in neurological diseases such as Multiple Sclerosis [2]. Intriguingly, increased iron content goes not only along with a remarkable signal loss in T2-weighted sequences (i.e. T2-hypointensity) but also blurs the differences in signal intensities between GM and white matter (WM) in T1-weighted sequences. Our MRI data sets included three sequences: gradient-echo T1-weighted and two different T2-weighted images. We decided to include all three sequences as their information is complementary. T1-weighted sequences are suitable for well established normalization pipelines. Besides the sensitivity to T2-hypointensity, one of the T2-weighted sequences was of high contrast but low image definition (FLAIR), while the other was of low contrast but high image definition (T2-weighted). Further, accessibility of these data to scientific investigations has been desirable as this MRI protocol has been used in routine clinical practice so that a still growing data base, including several large patient groups, has been available at our institution and cooperating institutions. Besides the fact that initially non-Bayesian approaches had failed with regard to both segmentation [3] and statistical analysis [4], we decided to develop algorithms based on Bayesian inference as some inherent features may be advantageous for preprocessing and statistical analysis of structural neuroimaging data [5]. For example, more realistic modeling of complex data is possible by incorporation of prior knowledge, and results do not have to be corrected for multiple statistical tests *post hoc.*

First, we developed a segmentation algorithm for the localization of T2-hypointensities by using outlier detection based on model checking techniques within a Bayesian mixture model. We used Markov Chain Monte Carlo (MCMC) methods for both model fitting and checking, as they allowed to incorporate not only the uncertainty of the data but also the uncertainty of model parameters, which often leads to results that are more realistic than those based on point estimates only [6]. The result of this segmentation tool was validated by simulated data and by visual inspection through relating segmented T2-hypointensities to anatomical GM regions known to contain iron above average.

Second, we adapted a Bayesian voxel-wise regression model that included spatial information during the estimation step via smoothness priors to mitigate the necessity to smooth images before statistical analysis. As in the mixture model and suggested earlier [7], [8], we also used MCMC methods for model fitting. Here, we had to adapt and extend earlier approaches designed for functional MRI (fMRI) data. Either those models [9], [10] were designed for first level analyses requiring an autoregressive component of the data so that they could not be applied directly to structural MRI data, or second level models did not account for the spatial structure of voxels so that images still had to be smoothed prior to statistical analysis. For validation, we used simulated data and data from healthy controls. We compared the results derived from our approach to those derived from standard software using either frequentist or Bayesian inference.

## Materials and Methods

### Subjects

MRI scans were obtained from 27 subjects (female, 17; age in years: range, 20–58; median, 29; mean standard deviation, 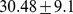) that had served as healthy controls in an MRI study (Departments of Neurology and Neuroradiology, Technische Universität München, Munich, Germany). Beforehand, written informed consent was obtained after description of the study to the subjects. The study was approved by the ethics committee of the medical faculty of the Technische Universität München, Munich, Germany, and performed in accord with the Declaration of Helsinki.

### Magnetic resonance imaging

All brain images were acquired on the same 3 Tesla scanner (Achieva, Philips, Netherlands). A three-dimensional (3D) GRE T1-weighted sequence (orientation, 170 contiguous sagittal 1 mm slices; field of view, 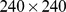 mm^2^; voxel size, 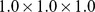 mm^3^; repetition time (TR), 9 ms; echo time (TE), 4 ms), 3D T2-weighted sequence (orientation, 144 contiguous sagittal 1.5 mm slices; field of view, 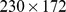 mm^2^; voxel size, 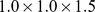 mm^3^; TR, 4000 ms; TE, 35 ms) and a 3D FLAIR sequence (orientation, 144 contiguous axial 1.5 mm slices; field of view, 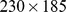 mm^2^; voxel size, 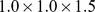 mm^3^; TR, 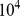 ms; TE, 140 ms; inversion time, 2750 ms) were used.

### Preprocessing

**Figure 1:**
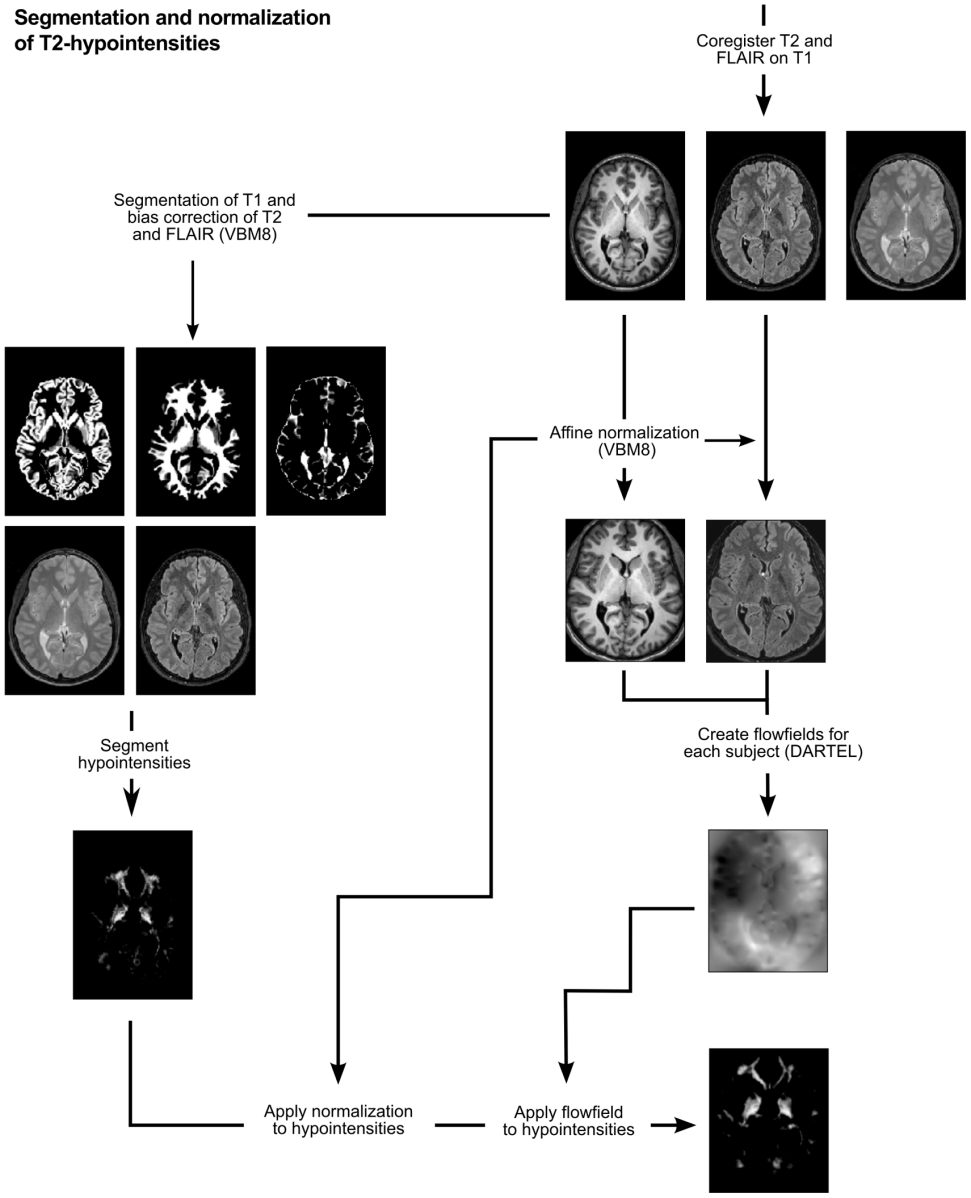
Segmentation and normalization of T2-hypointensity. T2-weighted and FLAIR images are first coregistered to the T1-weighted images and then prepared for the segmentation of T2-hypointensities, which includes correction of T2-weighted and FLAIR images for magnetic field inhomogeneity by VBM8 and segmentation of T1-weighted images into the tissue classes of GM, WM, and CSF. These images are then used to segment hypointensities. The resulting T2-hypointensity images are normalized in two steps: First, T1-weighted images are affine normalized and respective parameters applied to FLAIR and T2-hypointensity images. Second, affine normalized T1-weighted and FLAIR images of all subjects are used to produce individual flow fields by DARTEL; these flow fiields are then applied to T2-hypointensity images.

In this section, we describe preprocessing steps of our data with freely available software. SPM8 (http://www.fil.ion.ucl.ac.uk/spm) and its VBM8 toolbox (http://dbm.neuro.uni-jena.de/vbm) were used. An overview is given in Figure 1. First, T2-weighted and FLAIR images are coregistered to the T1-weighted images in the original (‘native’) space. These images are then prepared for the segmentation of T2-hypointensities, which includes correction of T2-weighted and FLAIR images for magnetic field inhomogeneity by VBM8 (function ‘estimate and write’, default option; output option, ‘bias corrected’ and ‘native space’) and segmentation of T1-weighted images into the tissue classes of GM, WM, and cerebrospinal fluid (CSF) (function ‘estimate and write’, default option) as this information is necessary to segment hypointensities as explained in the next section. The resulting images of segmented T2-hypointensity (T2-hypointensity images) are normalized in two steps: First, T1-weighted images are affine normalized and respective parameters applied to FLAIR and T2-hypointensity images. Second, affine normalized T1-weighted and FLAIR images of all subjects are used to produce individual flow fields by high-dimensional warping as implemented in SPM8 (‘DARTEL’, [11]). Each sequence was entered as one class to improve normalization by simultaneously accounting for information of both sequences and, hence, also accounting for regional T2-hypointensity. The resulting normalized images of segmented T2-hypointensity were analyzed for age-related effects by our voxel-wise regression approach.

### Segmentation of T2-hypointensity

This section describes the first objective of this study, namely the segmentation of T2-hypointensities. It contains four subsections: 1) Introduction of the Bayesian mixture model, 2) estimation of model parameters by MCMC methods, 3) detection of T2-hypointensities by Posterior Predictive Checks (PPC), and 4) validation through simulated and real data.

#### Bayesian mixture model

Here we present a Bayesian mixture model [12], [13] that is used to fit the intensities of the two T2-weighted sequences (T2-weighted and FLAIR). It is first explained in general terms and later adjusted for the data at hand.

In a mixture model, it is assumed that the observed data 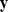 can be divided into 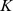 unobserved classes. The components of the data vector are vectors of dimension 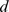, i.e. 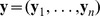 with 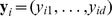. In order to derive the likelihood for the data conditioned onto the respective class, a class indicator 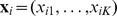 for each observation 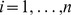 is introduced, where the 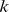
th element of 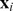 is set to 1 if voxel 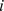 belongs to class 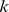. In most applications, the indicators 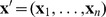 are missing and it is the purpose of a mixture model to estimate these missing observations. Conditioned on the class indicator 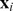, the distribution of observation 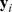 is given by
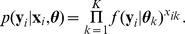



Commonly, it is assumed that the mixture components 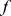 are all from the same parametric family and differ only by their parameters 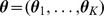, which is not a real restriction but simplifies notation. For a given mixture distribution 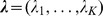 with 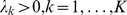 and 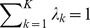, the distribution of the unknown class indicators is a multinomial distribution:
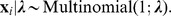



Thus, the joint distribution of the observed data 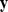 and the missing label indicators 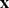 can be written as
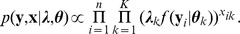



In order to perform inference, prior distributions have to be specified for 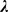 and the parameters in 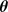. One possible choice for 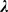 is its natural conjugate, that is a Dirichlet distribution with hyperparameter 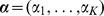 [14]. For 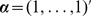 this prior can be seen as non-informative. The choice of priors for the parameters in 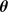 depends on the choice of the mixture components 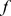.

In summary, the joint posterior distribution of all unknown parameters is given by
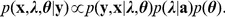
(1)


This distribution is analytically not feasible but MCMC methods can be used to simulate this posterior.

For the segmentation of our data, we adjusted the mixture model as two different T2-weighted sequences were available, which were either of high image definition but low contrast (T2-weighted), or of low image definition but high contrast (FLAIR). Aiming at the best possible segmentation of T2-hypointensity, we utilized both pieces of information in order to reduce the effect of sequence-specific artifacts. Yet, as the tissue class of CSF is also hypointense in FLAIR sequences, we excluded voxels representing CSF according to the segmented images of the T1-weighted sequence by VBM8, i.e. the number of classes 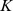 in our study is two. Intriguingly, the distinction between GM and WM based on T1-weighted images is problematic particularly in the areas of interest in this study, namely T2-hypointense GM regions, since they have an increased iron content, which increases the T1-weighted signal and hence shifts its intensity from GM towards WM [15]. Against this backdrop, we decided to model T2-hypointensity for the two tissue classes of GM and WM separately but to generate a single image of T2-hypointensity across both GM and WM.

With the restrictions outlined above, the probabilities regarding the brain tissue classes (GM and WM) are already known from the segmentation of the T1-weighted images and can therefore used as additional prior information. Let 
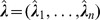
 with 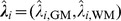 denote these probabilities, then it is assumed that the class indicators are multinomial distributed with parameter 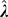. Note that VBM8 incorporates spatial prior information of adjacent voxels into the segmentation estimation by a Markov Random Field [16]. Therefore, we did not further account for spatial correlation at this step. However, we will later consider neighbouring information during outlier detection.

Finally, because the marginal histograms of the two remaining tissue classes are considerably skewed, we use two bivariate mixture models for the mixture components and therefore introduce the subclass indicators 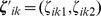 with
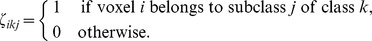



In similarity to the class indicators, the subclass indicators 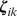 follow a multinomial distribution with mixture distribution 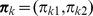, which leads to the following joint distribution for the observed intensities and the missing class and subclass indicators
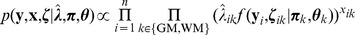
(2)with mixture components defined as



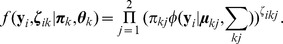



Here, 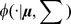 is the density function of the multivariate (bivariate, in this case) normal distribution with mean 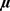 and covariance matrix 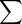 and all subclass indicators are collected in 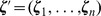 with 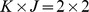 elements 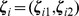.

Since the size of the data is quite large (over 1.1 million relevant voxels for each brain), the influence of prior distributions on the parameters of the mixture components as well as the mixture distribution will be limited and the inference will be dominated by the likelihood. We therefore choose non-informative flat prior distributions for these parameters. In detail, we use independent Dirichlet priors for the mixture distributions 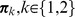 with hyperparameters set to 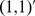 and an independent Jeffrey's prior for 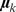 and 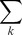, that is 
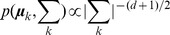
. This does not only reflect our lack of knowledge about these parameters but also simplifies the MCMC algorithm. In summary, the joint posterior distribution of all unknown parameters is given by
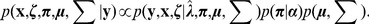
(3)


#### Parameter estimation

For the proposed model, all full conditional distributions can be derived in closed form. As this part is not crucial for understanding the general segmentation approach, the reader may skip to the next subsection.

The full conditional distributions for the subclass indicators of voxel 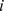 that belongs to class 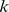 can be derived from the joint distribution of 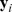 and 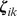:
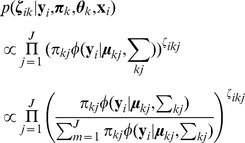



The last term is the core of a multinomial distribution with parameters
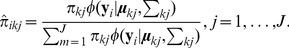
(4)


Since the Dirichlet prior is the natural conjugate for the parameters of a multinomial distribution, the full conditional for the mixture distribution 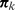 is a Dirichlet distribution with updated parameters 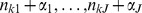, where 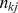 is the number of observations in subclass 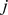 of class 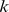.

For given class and subclass indicators, the parameters of the mixture components are updated for each class and subclass separately. For 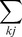 the marginal posterior under the proposed flat prior is an inverse Wishart distribution
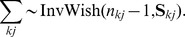
Here, 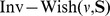 stands for the inverse Wishart distribution with 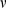 degrees of freedom and scale matrix 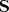. The matrix 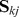 is the sample covariance matrix of the intensities in class 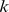 and subclass 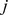




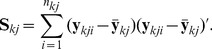
(5)The posterior for 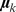 conditioned on 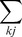 is then a multivariate normal distribution with mean equal to the mean of the intensities in class 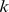 and subclass 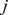, 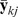, and covariance matrix 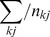.

In summary, the following Gibbs sampler can be used to simulate distribution (3):

1. Initialize the class and subclass indicators 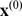 and 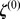.
2. For 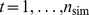 repeat the following steps:
  - For current 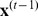 and 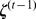 calculate the sample covariance matrix 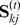 for 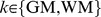 and 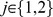 according to (5) and draw 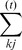 from an inverse Wishart distribution with scale matrix 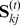 and 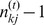 degrees of freedom.
  - For 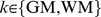 and 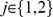 draw 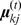 from a normal distribution with mean 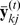 and covariance matrix 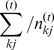.
  - For 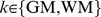 draw 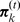 from a Dirichlet distribution with parameters (
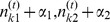
).
  - For 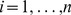 and 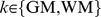 draw 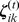 from a multinomial distribution with parameters 
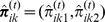
 according to (4).
  - For 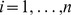 draw 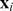 from a multinomial distribution with parameters 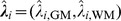.
3. After discarding the realizations of an initial burn-in phase, the remaining samples can be considered as dependent samples of the joint posterior (3).

**Figure 2:**
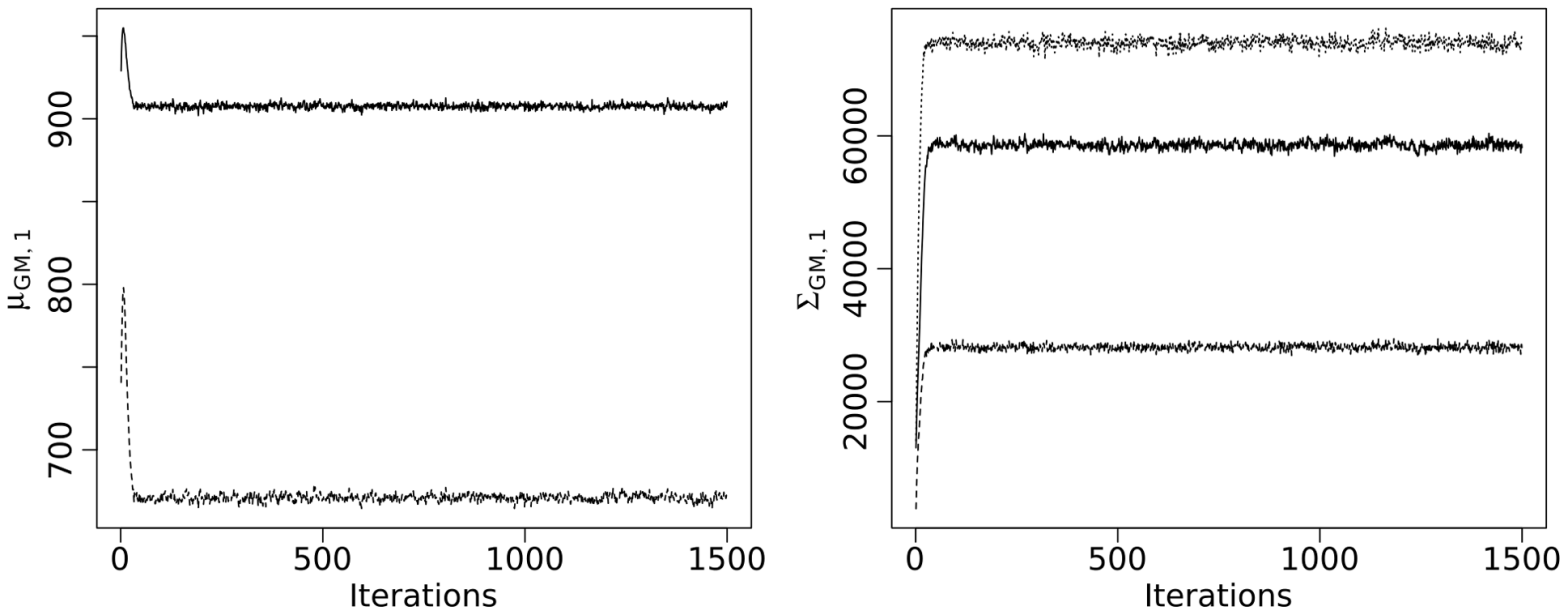
Trace plots for the Gibbs sampler of the mixture model for T2-hypointensity segmentation of one randomly chosen subject. Components of 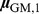 (left) and of 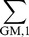 (right). See text for details.

For each subject, three parallel chains of length 1500 were calculated by the Gibbs Sampler described above. Class and subclass indicators were initialized at random. Figure 2 shows trace plots of such a chain for one randomly chosen subject. The left panel displays the components of 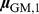 and the right panel those of 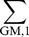. As it can be seen, mixing of chains is quite good and label switching [13] does not occur. For the calculation of T2-hypointensities, we discarded the initial 500 draws and kept every second sample. For the remaining draws we calculated Gelman and Rubin's potential scale reduction factor [17] for the mean of the parameters of the mixture components and the mixture distributions. In all cases, the value was nearly indistinguishable from 1 indicating that the simulation converged to the target distribution [17].

#### Posterior predictive checks

Fitting the model will yield 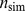 realizations of the posterior distribution. Denote these samples by 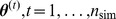. Those samples can be used to perform PPC in order to check the fit of the model [18], [19] or to identify outliers, as explained next. The basis for PPC are replicated samples (‘fake’ data) 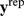 of the observed data 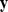 according to the posterior predictive distribution (PPD):
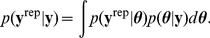
(6)


**Figure 3:**
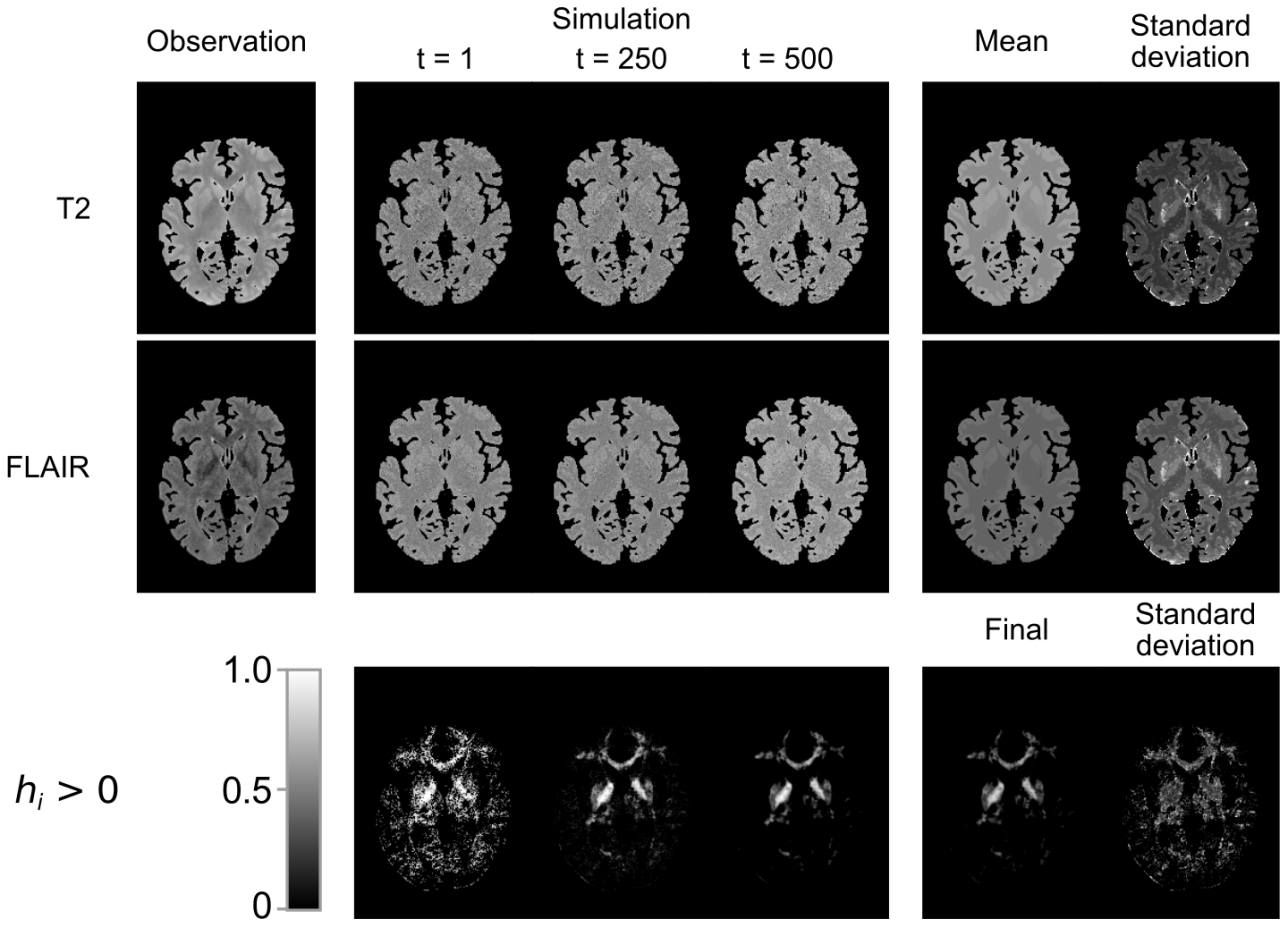
Simulation and outlier detection of T2-hypointensity. Images were derived from a randomly chosen subject. On the left, a normalized T2-weighted (top) and a normalized FLAIR (bottom) image is shown (only gray and white matter). Three examples of respective simulated images and their means and standard deviations are shown in the middle and on the right, respectively. In the lower row, respective positive values of the hypointensity score, derived from both T2-weighted and FLAIR images, as well as their final image and standard deviation are shown and gray-scaled according to the bar in the lower left. See text for details.

To generate samples 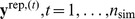 out of this distribution, one proceeds as follows: For each realization of the unknown parameters generate 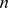 samples according to the likelihood. In the case of the mixture model explained above, we generate 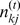 samples (according to the actual label configuration 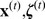) of a bivariate normal distribution with parameters 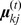 and 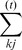. Figure 3 illustrates this procedure for a randomly chosen subject. The first row shows a slice of the observed T2-intensities followed by three simulated slices. The last panel in the first row displays the mean and standard deviation of the simulated intensities. The second row displays the same information for the same slices of the FLAIR-image. In both cases it can be seen that hypointense structures visible in the observed images are not present in the calculated mean images. This illustrates that we can detect T2-hypointensities by comparing the replicated images to the observed image.

In general, once the replicated data sets are available, they can serve to measure the discrepancy between the model and observed data by analyzing test quantities, or general discrepancy measures 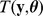. This discrepancy measure is calculated for the observed and replicated data. It can be any kind of scalar summary of the data. The calculated discrepancies may be displayed graphically to perform visual model checks or by using Bayesian posterior predictive 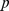-values [6]. For segmentation, we record if the replicated intensity of voxel 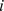 is greater or equal than its observed intensity, hence, we choose 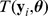 to be the intensity value itself. We denote this by
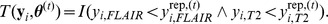



Based on this information, we iteratively build the hypointensity score 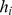 for each voxel 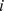 by applying the following formula
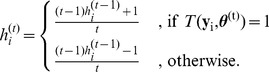
(7)


In this form, the final hypointensity score is simply the mean of minus and plus ones, where plus one results if the replicated intensity is greater than the observed one. Thus the intensity score takes values between −1 and 1 with positive voxels indicating more hypointensity and negative values less hypointensity. To account for spatial dependencies between adjacent voxels, we expand equation (7) to
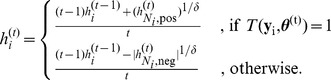



Here, 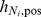 is the mean of all neighboring intensity scores that are positive. Likewise, 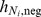 is the mean of all neighboring intensity scores that are negative. The parameter 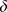 controls the influence of neighboring intensity scores on 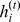.

The last row in Figure 3 displays positive values of 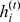 for three different iterations and the final segmentation along with its standard deviation. According to the validation, the parameter 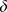 was set to 1.4. See next paragraph for details.

#### Validation

**Figure 4:**
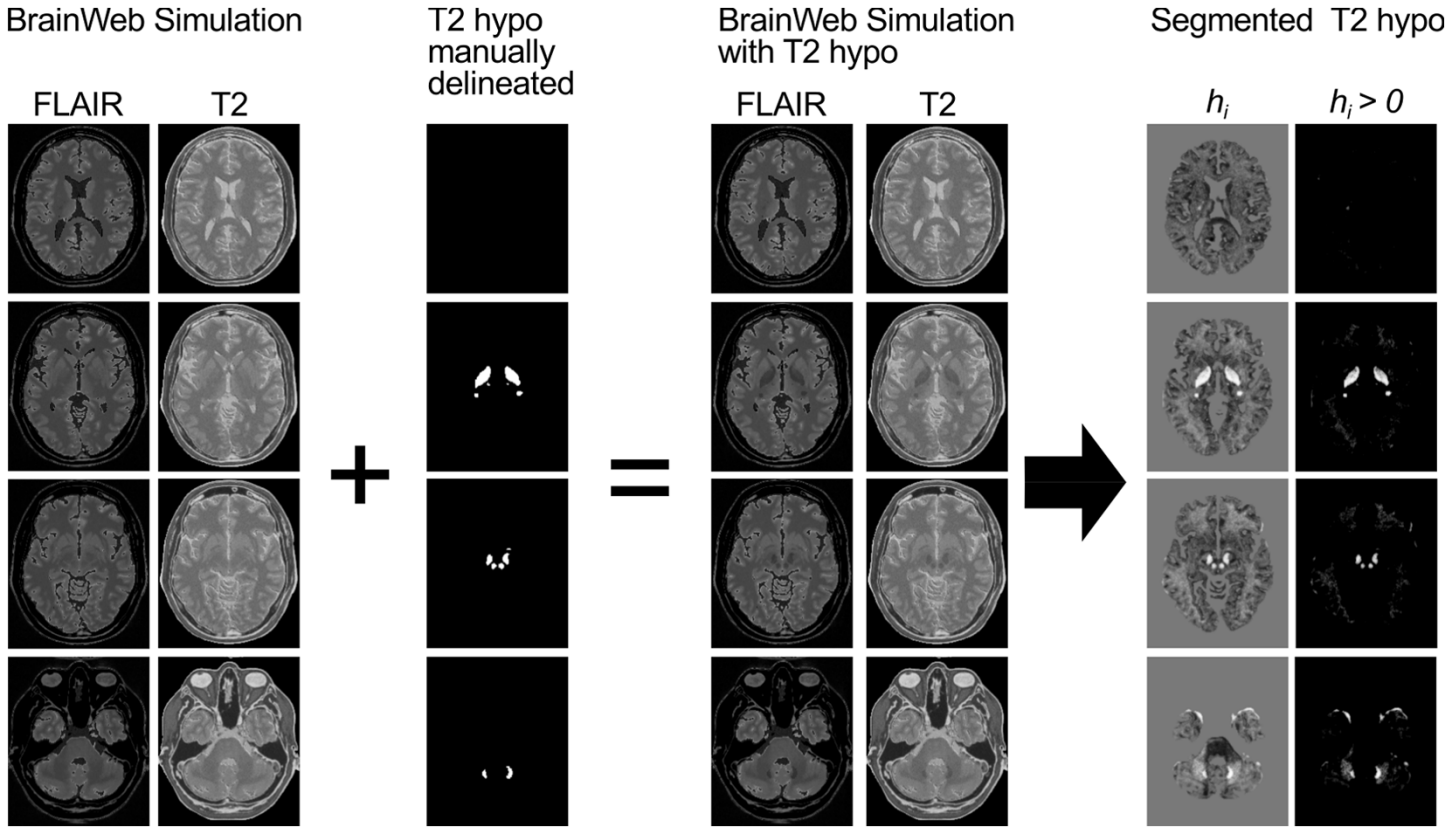
Segmentation of simulated T2-hypointensities. Manually delineated T2-hypointensities were added as an extra class to BrainWebs discrete anatomical model. This way, T1-weighted, T2-weighted and FLAIR images were simulated. Hypointensities were then segmented from the simulated images.

First, we validated our segmentation procedure by a simulation study. Accounting for the lack of a commonly accepted gold standard, we manually labeled hypointense regions that are visible in the mean FLAIR images of our healthy controls. Before averaging, images were normalized by the use of the deformation field derived from standard normalization of T1-weighted images as implemented in VBM8. We then added this binary label as an extra class to BrainWeb's (http://brainweb.bic.mni.mcgill.ca/brainweb/) discrete anatomical model [20] and simulated T1-weighted, T2-weighted and FLAIR images by BrainWeb's MRI simulator [21]. Selected slices of the simulated T2-weighted and FLAIR images without and with T2-hypointensities as well as of the binary label are shown in Figure 4. We applied our algorithm to the simulated images with values of 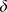 ranging from 1 to 4 with an increment of 0.05 and determined the optimal value by calculating the Dice coefficient (DC, [22], [23]). We also considered the influence of different values for the binary threshold ranging from 0 to 1 with an increment of 0.05 for each value of 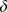. Beyond the simulation study, we biologically validated the algorithm by visually comparing the segmented T2-hypointensity images with both the T2-weighted images and the FLAIR images. To evaluate the effect of the incorporation of both T2-weighted sequences (T2-weighted and FLAIR) into the segmentation by the mixture model, we repeated segmentation of T2-hypointensity with an adapted version of the model twice after having subjected either only the T2-weighted or only the FLAIR images.

### Bayesian voxel-wise regression with smoothness priors

This section describes the second objective of this study, namely the adaption of a voxel-wise linear regression model. It contains four subsections: 1) introduction of the model, 2) parameter estimation, 3) calculation of posterior probability maps, and 4) validation.

#### Description of the model

It is still a challenging question in neuroimaging, how to handle spatial correlations in the data, alongside the ideas that we expect effects of interest to occur in clusters of voxels and that models accounting for these dependencies are likely to be more robust. Existing frequentist methods use a combination of pre-smoothing and spatial statistics based on Random Field Theory. However, these frequentist approaches rely on the subjective selection of a number of parameters, such as the amount of spatial smoothing to impose, and the choice of cluster forming thresholds. Of note, Bayesian inference offers the possibility of a solution to these problems via the integration of the dependency among adjacent voxels into the regression model itself [5]. Those approaches were formulated previously in the context of first level fMRI analyses [7], [8] but not for second level analyses as necessary for structural MRI data so that we had to adopt previously proposed approaches.

Let 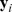 denote the 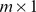 vector of responses of voxel 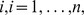 for the 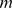 subjects, the regression model for the 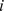
th voxel can be written as
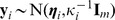
where 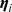 denotes the linear predictor, 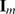 the 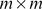 identity matrix and 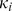 the unknown precision parameter, i.e. the inverse variance. The linear predictor has the form



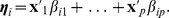



In this notation, the 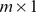 vector 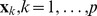 collects the values of the 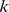
th covariate for all subjects and 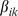 represents the corresponding unknown regression coefficient. In most applications, the first covariate is 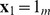 and thus 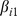 is the intercept in the model for the 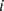
th voxel. In the study presented here the linear predictor consists of an intercept and main effects of age and sex.

By defining 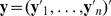 and 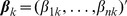 for 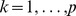, the 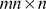 matrix
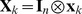
and the 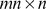 diagonal precision matrix



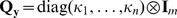
the complete model can be written in compact matrix notation as



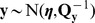



with
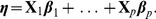
(8)


We use independent Gaussian Markov Random Field (GMRF, [24]) priors for the regression coefficients as they are commonly used in neuroimaging in order to account for the spatial structure of images, see for example [25], [10] and [26]. We therefore have
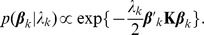
Here, 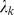 is a precision parameter (inverse variance) and 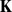 is a structure matrix. Whereas 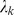 operates as a smoothness parameter that is estimated by the data, the matrix 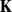 accounts for spatial dependencies between the regression coefficients. The elements of 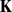 are



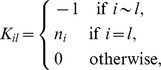
where the number of voxels in the neighborhood of voxel 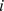 is denoted by 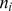 and 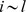 stands for all voxels 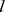 that share a common border with voxel 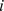, that is we use a first order neighborhood consisting of the six nearest neighbors. One advantage of such a prior is that it acts like a smoothness prior. To show this, the full conditional of 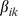, given all the other values of 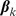, can be written as



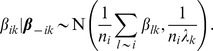



Thus, the conditional prior corresponds to a normal distribution with expectation equal to the mean of the effects of neighboring voxels and precision proportional to the number of neighboring voxels and precision 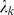.

To perform fully Bayesian inference, priors for the precision parameters 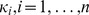 and 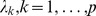 have to be chosen. We chose independent Gamma distributions with hyperparameters 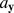 and 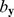 for the precisions of 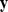 and 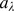 and 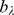 for the precisions of the regression coefficients. By adopting small values for the hyperparameters, for example 0.1, 0.01 or 0.001, one obtains ‘diffuse’ priors for the precision parameters.

In our study, response values of the voxel-wise regression model are the segmented hypointensities. Besides an intercept and the effect of age, sex is included as a dummy-coded factor (0 = male, 1 = female) yielding the model
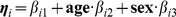
(9)for voxel 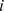. Here, age and sex are the vectors of age and sex, respectively.

#### Parameter estimation

To obtain samples from the joint posterior
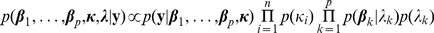
(10)a Gibbs sampler can be used. As this part is not crucial for understanding the adoption of our voxel-wise linear regression model, the reader may skip to the next subsection.

To obtain the full conditionals for 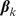, let 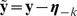, where 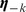 is the linear predictor (8) without the 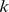
th term. Then, the full conditional for 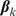 is given by
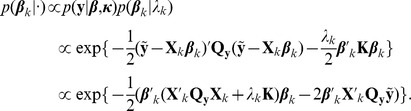



By completing squares, we obtain
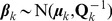
(11)with



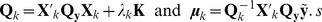
(12)The full conditional for smoothness parameter 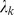 are obtained by
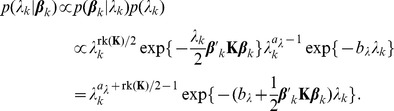



Thus, the full conditional for 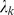 is a Gamma distribution with updated parameters
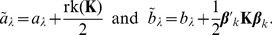
(13)


With a similar calculation, it can be shown that the full conditional for the precision parameter of voxel 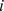, 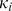, follows a Gamma distribution with updated parameters
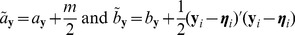
(14)thus, the precision parameters 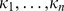 can be updated for each voxel independently.

1. Initialize the precision parameters 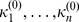 and 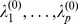 as well as the regressions coefficients 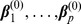.
2. For 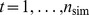 repeat the following steps:
  - For current 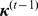 calculate 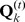 and 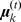 for 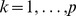 according to (12) and draw 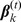 from a multivariate normal distribution with mean 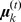 and precision matrix 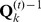.
  - For 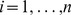 draw 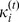 from a Gamma distribution with shape and rate parameters according to (14).
  - For 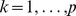 draw 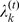 from a Gamma distribution with shape and rate parameters according to (13).
3. After discarding the realizations of an initial burn-in phase the remaining samples can be considered as dependent realizations of the joint posterior (10).

By sampling 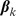 from its full conditional, we make use of the independence structure that is imposed by the voxel layout. To be more precise, we split all voxels in two sets of independent voxels according to the first order neighborhood. This has the advantage that, conditioned on each other, the precision matrix of the full conditional for each set is diagonal. Thus, calculating the corresponding Cholesky triangle is not necessary anymore and sampling of 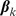 becomes feasible while still considering the full covariance structure [24].

Hyperparameters for the precision and smoothness parameters are set in accordance with [7] to 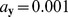 and 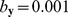 and to 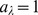 and 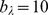.

**Figure 5:**
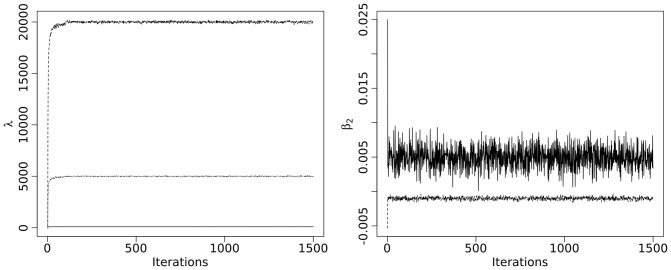
Trace plots of the voxel-wise regression model. Precision parameters (left) and main effect of age for two selected voxels (right). Corresponding MNI coordinates are 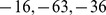 (top) and 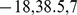 (bottom).

To fit model (9) to the data, three parallel chains of length 1500 were calculated. Starting points were generated randomly in the interval 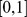 for precision parameters and in the interval 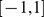 for regression coefficients. Trace plots of the precision parameters 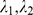 and 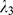 of one chain are shown in the left panel of Figure 5 and of two selected voxels of 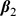 in the right panel of this figure. MNI coordinates of voxels are 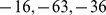 (top) and 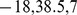 (bottom). As for the mixture model, the initial 500 draws were discarded and additional 500 draws of each chain were saved. Again, we calculated Gelman and Rubin's potential scale reduction factor for the mean of the precision parameters. In all cases, it can be assumed that the simulation converged to the target distribution.

#### Calculation of posterior probability maps

Results for the regression coefficients can be displayed in different ways. In order to compare the results with those derived from an already existing implementation, we calculated posterior probability maps. Usually, this is achieved by computing 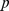-values based on the analytical marginal posterior distributions [27]. Here, we estimate the probability 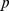-values of a positive or negative effect of the predictors by the proportion of the corresponding MCMC draws that lie above or below zero, respectively.

#### Validation

First, we validated our voxel-wise regression model by a simulation study. In accordance with Penny et al. [10], we generated a two-dimensional 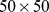 pixel image of regression coefficients that contains Gaussian blobs. It was created by placing circular effect patterns with heights ranging from 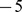 to 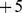 on seven different locations. Radii of effects ranged from 1 to 4 pixels. Gaussian blobs were obtained by smoothing these effects individually with Gaussian kernels having different full width at half maximum (FWHM) ranging from 1 to 8 pixels. In order to simulate observation images, we generated values for one metric covariate at random between 0 and 1 and multiplied the coefficient image with those values. Finally, we randomly generated a precision parameter for each pixel using a gamma distribution with shape and scale parameters set to 2. Gaussian noise with precision set to these parameters was added to the multiplied images of regression coefficients. This way, we generated 30 ‘fake’ observations. The parameter image was estimated by the presented approach and by SPM8 (both standard frequentist and Bayesian implementation) after smoothing the observation images with Gaussian kernels of 0 and 4 pixels. Results were compared by visual inspection and by calculating the mean squared error (MSE) between the true and the estimated coefficient images.

Second, we biologically validated our model by analyzing our normalized segmented T2-hypointensity images for age-related effects. This validation is justified as it is commonly accepted that the loss in the T2-weighted signal within the most T2-hypointense GM areas is due to an increased iron content, which is not only related to neurodegeneration but also to normal aging [15], [28], [29]. We compared the results derived from our approach to those derived from SPM8. Yet, we will not report the results of the Bayesian approach implemented in SPM8, which yielded implausible results. This could be replicated with simulated data by drastically increasing the ratio between voxels without an effect and those with an effect. We reported this problem, which is intended to be fixed. To compare our approach to the frequentist approach in SPM8 we applied different smoothing kernels, namely a Gaussian kernel of 0, 4, and 8 mm FWHM. As significance thresholds, we chose a posterior probability of 0.95 or, correspondingly, a false discovery rate (FDR) of 0.05 [27]. In all cases, the effect size threshold was set to zero. We restricted our analyses to voxels with a mean hypointensity score of greater than 0.25.

### Software

We implemented both presented approaches in pure MATLAB (http://www.mathworks.de/products/matlab/) code. Segmentation of one subject took about 20 minutes with a 3.2 GHz processor. On the same machine, one chain for the voxel-wise regression model could be obtained within six hours requiring about 20 GB RAM.

## Results

### Segmentation of T2-hypointensities

Visual inspection of the segmented T2-hypointensities of simulated data showed that T2-hypointense regions were reliably detected. Spurred false positives occurred at the border to CSF. The highest DC value (0.754) was observed for a 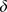 of 1.4 and for the binary threshold of 0.45. This excellent similarity measure [30], [31] was robust as indicated by DC values greater than 0.7 after changing 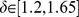 and the binary threshold in 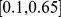. Selected slices of the hypointensity score for the simulated images and 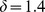 are shown in Figure 4. For further analyses, we chose a 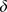 value of 1.4.

**Figure 6:**
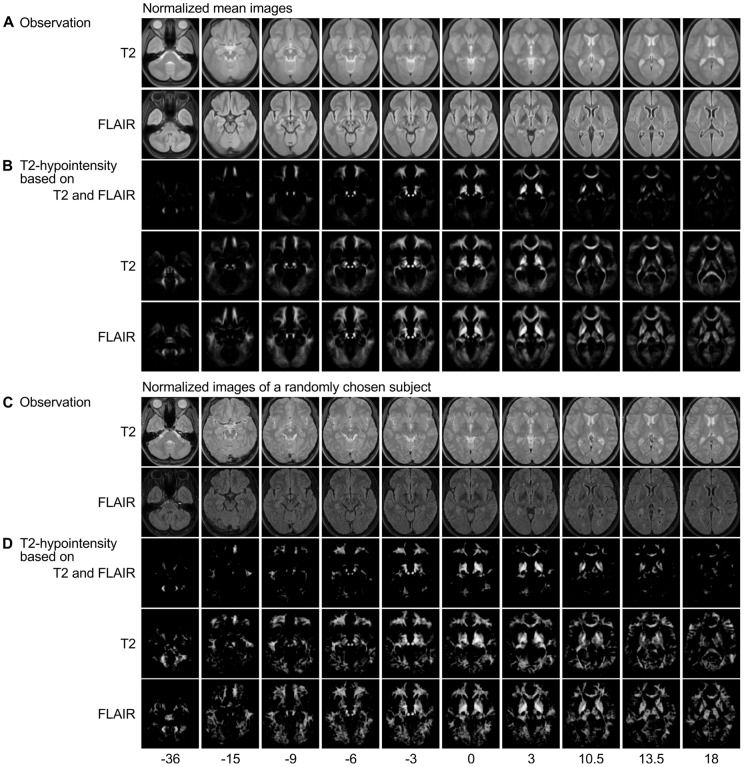
Segmented T2-hypointensity. A) Axial slices of normalized mean images (T2-weighted and FLAIR) are shown. B) Corresponding axial slices of segmented T2-hypointensity are shown (upper row, based on both T2-weighted and FLAIR images; middle row, based only on T2-weighted images; lower row, based only on FLAIR images). C and D) Information of a randomly chosen subject is given in analogy to panels A and B; for better illustration, normalized images are shown, although the algorithm operates in the original (native) space. See text for details.

Segmentation of T2-hypointensities in healthy controls is displayed in Figure 6. The first five rows show the mean images derived from the whole group, the last five rows show the images derived from a randomly chosen subject. Structures known to be T2-hypointense are clearly visible, that is the globus pallidus, substantia nigra, red nucleus, and dentate nucleus. Further, the use of both T2-weighted sequences (T2-weighted and FLAIR) resulted in more accurate segmentation than the use of only one sequence.

### Voxelwise regression model

**Figure 7:**
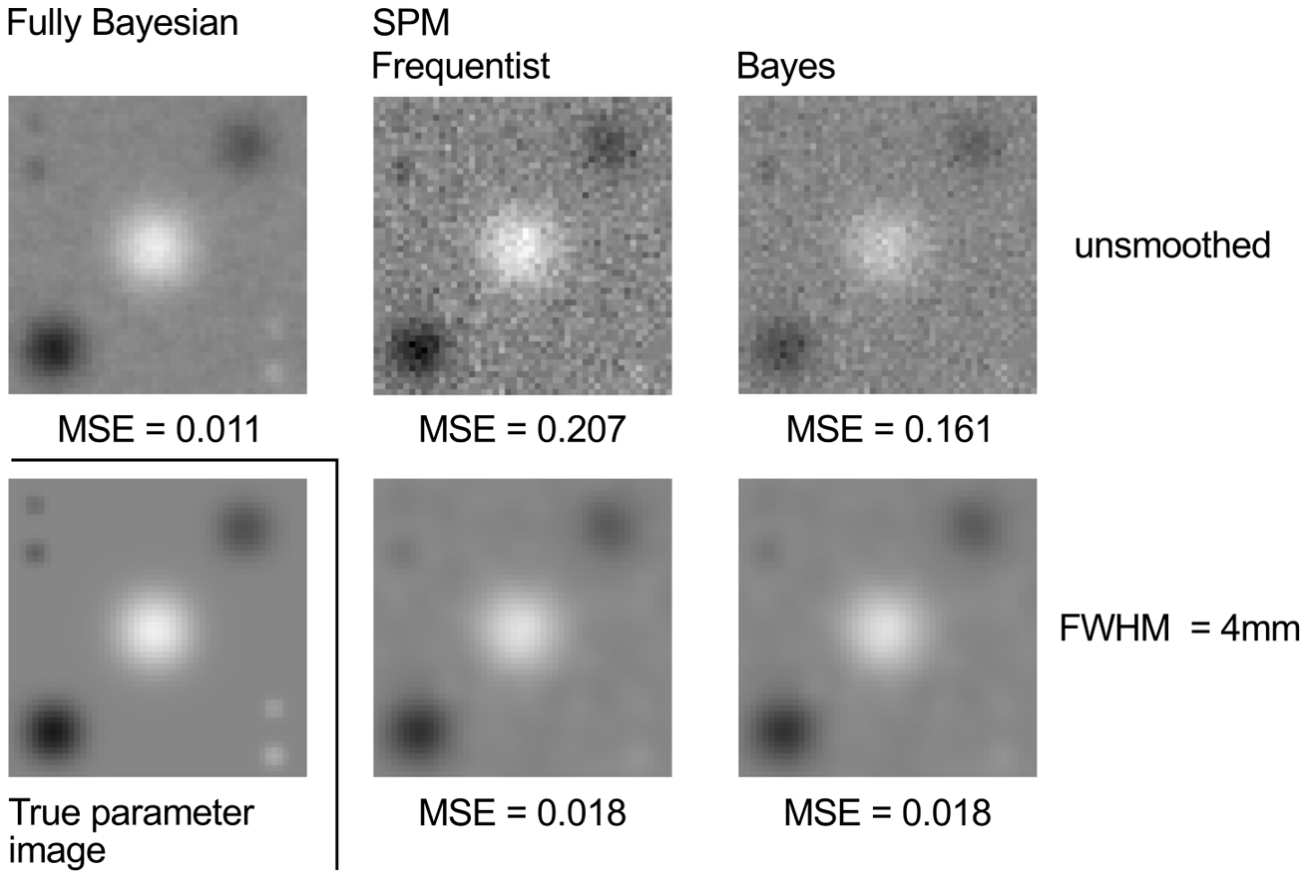
Estimated regression coefficients of the simulated data. Posterior mean image for unsmoothed data of the approach proposed in this paper is shown in the upper left corner. Results of SPM's frequentist and Bayesian implementation are shown in the second and third column for unsmoothed (upper row) and smoothed (lower row) data, respectively. The true parameter image is shown in the lower left corner. The approach proposed in this paper performs best as demonstrated by the MSE and by visual inspection.

Estimated regression coefficients of our simulated data are shown in Figure 7. The approach proposed in this paper performs best as demonstrated by the MSE and by visual inspection. While all blobs of the true coefficient image are visible in our estimation, both SPM's frequentist and Bayesian implementation fail to detect smaller effects.

**Figure 8:**
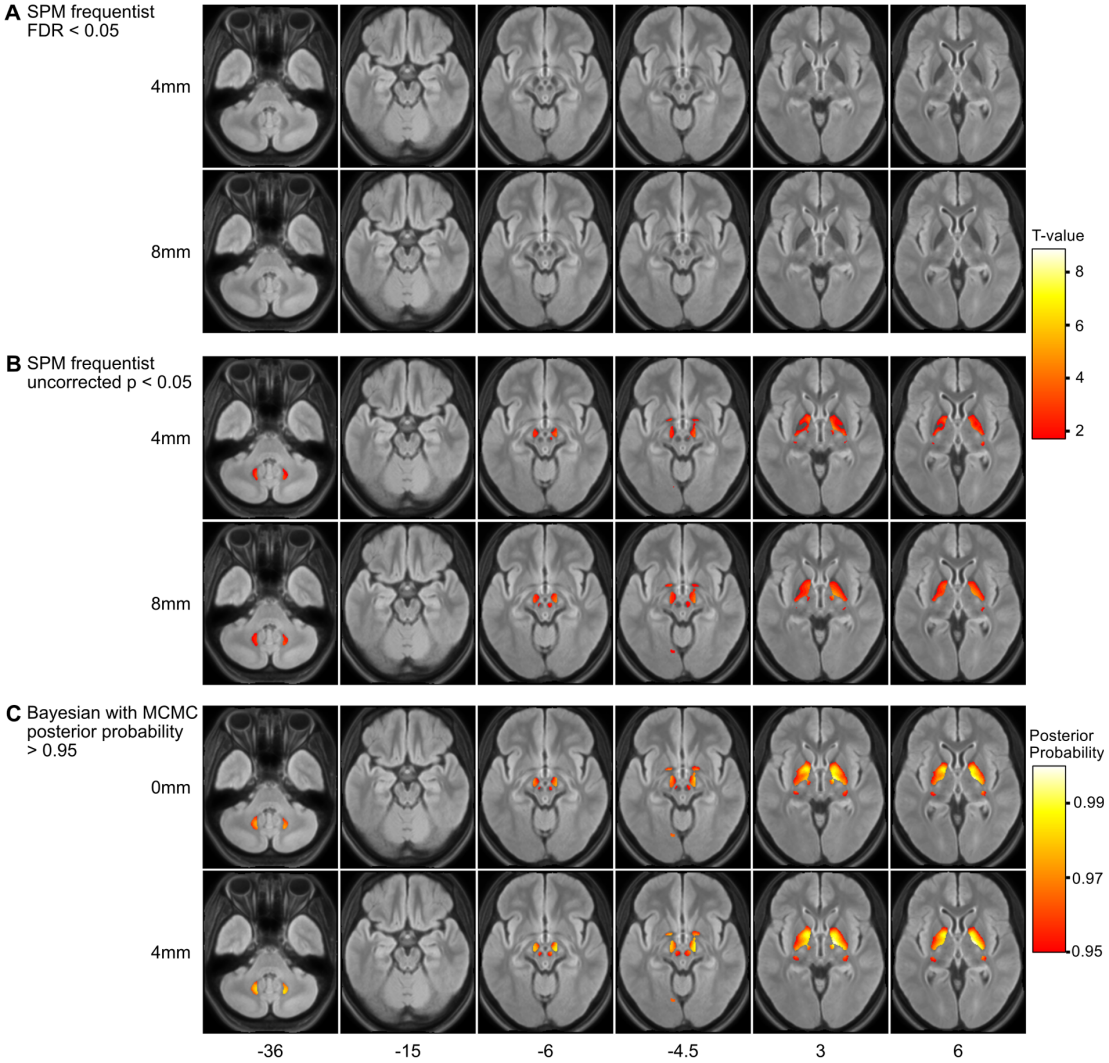
Effect of age on T2-hypointensity. Increasing T2-hypointensity with increasing age is projected onto the mean normalized FLAIR image. Axial slices are indicated in the upper row. Significance is color-coded according to the 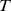-value (Panels A and B) and posterior probability (Panel C) as indicated by the bars on the right. A–B) Results derived from the frequentist approach as implemented in SPM8 are shown after application of different statistical thresholds (Panel A, false-discovery rate 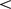
0.05; Panel B, uncorrected 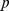-value 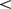
0.05) and different smoothing kernels (upper rows, 4 mm; lower rows, 8 mm. C) Fully Bayesian inference could not only identify the globus pallidus, substantia nigra, and red nucleus but also the dentate nucleus. This result was largely independent of smoothing although more voxels were identified after smoothing with 4 mm.

Within the GM of our healthy controls, we observed only T2-hypointensity that increased with increasing age. The results derived from different multiple linear regression models yielded different results, which will be described in correspondence to the number of identified voxels from low to high. The conventional frequentist approach did not yield any meaningful results neither at the pre-defined significant threshold nor at the voxel threshold of 0.05 family-wise error corrected (Figure 8, Panel A). Only after relaxing the voxel threshold to 0.05 uncorrected, we observed all expected GM regions, namely globus pallidus, substantia nigra, red nucleus, and dentate nucleus (Figure 5, Panel B). By the use of our fully Bayesian approach, we could not only identify the globus pallidus, substantia nigra, and red nucleus but also the dentate nucleus. This result was largely independent of smoothing although more voxels were identified after smoothing with 4 mm (Figure 5, Panel D).

## Discussion

In this work, we have developed and validated algorithms based on fully Bayesian inference to preprocess and statistically analyze structural MRI data. Separately for preprocessing and statistical analysis, we will discuss the rationale, realization and validation of our approaches. We will also acknowledge limitations of our work and outline room for improvement.

In the first part of our study, we developed a tool for segmentation of T2-hypointensity, which, to the best of our knowledge, is the first that utilizes PPC for outlier detection in the context of neuroimaging. The concept of PPC derives its flexibility from the possibility that any scalar summary of the data can be chosen for the discrepancy measure 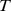 and that it can be applied to every model that has been fitted in a fully Bayesian way. In principle, simulation based model checking techniques can also be applied in the framework of non-Bayesian estimation methods [32] given that (asymptotic) distributions of model parameters can be obtained, for example, by standard errors of parameters. However, commonly used iterative algorithms, such as the expectation maximization algorithm, need to be extended to estimate standard errors, which has been regarded technically challenging [33]. Therefore, we decided to address the segmentation problem by PPC based on a fully-Bayesian approach. The resulting segmentation algorithm was fully automatic and operated across the whole brain. Influence of adjacent voxels during segmentation can be controlled by the 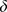-parameter. Further, no thresholds had to be chosen and user-defined regions of interest did not have to be defined. Moreover, the flexibility of the proposed mixture model enabled the incorporation of two different T2-weighted sequences, which clearly improved the precision of T2-hypointensity segmentation. As a result, all GM regions known to be T2-hypointense in healthy subjects were segmented reliably and almost exclusively in both simulated data and real data even at the single subject level. As T2-hypointense GM areas display an increased T1-weighted signal similar to that of WM, we were unable to clearly attribute T2-hypointensity to one of the two tissue classes through our model. Therefore, we included all brain parenchyma, namely GM and WM, in our segmentation. Accordingly, our tool also segmented WM areas. These areas, primarily the corpus callosum and frontal forceps, are known to contain tightly packed fibers so that segmentation of these WM areas can be attributed to the lowest T2-weighted WM signal of these regions [34]. Hence, segmentation of WM is inherent to our approach and biologically plausible. Moreover, the proposed mixture model may be extended in many further ways. For instance, different distributions can be chosen for the mixture components to obtain a better fit to particular structures of the intensity distribution. Further, prior information for the location of T2-hypointensity may be constructed and used within the segmentation step. Although our tool accounts for the information of three different MRI sequences, the approach is still hierarchical as information of the T1-weighted image constitutes the basis for the segmentation of the two T2-weighted images. However, a truly multimodal segmentation that simultaneously accounts for all available information is likely to be advantageous over our algorithm.

In the second part of our study, we adapted a voxel-wise linear regression model through Bayesian inference. In contrast to SPM8, which applies global shrinkage priors [27], our approach accounts for the spatial dependency of voxels within the estimation procedure by the use of GMRF priors. Further, smoothness parameters are estimated from the data at hand by MCMC methods. We expected our approach to be advantageous over conventional frequentist and available Bayesian approaches for three reasons. First, eliminating the necessity for *post hoc* correction for multiple comparisons should increase statistical power compared to conventional frequentist approaches. Second, accounting for the spatial dependency of voxels within the estimation procedure mitigates the necessity to smooth images in order to increase the signal to noise ratio [10]. The spatial dependency of voxels has not been included in available frequentist approaches (apart from smoothing) and only in some available Bayesian approaches. For example in SPM8, the spatial dependency of voxels is considered within the estimation procedure for analysis of fMRI time series at the first level [10] but not for analyses at the second level [27]. Yet, we did not expect our results to be completely independent of smoothing, since it also compensates for imperfect coregistration. Third, more accurate approximation of posterior distributions by MCMC methods should increase both sensitivity and specificity compared to available Bayesian approaches. Of note, all three assumptions comply with the results of our validation through simulated data. Further, biological validation by analysis of age-related T2-hypointensity yielded plausible results. Compatible with increasing iron content with increasing age [35]–[37], we found increasing T2-hypointensity with increasing age exclusively, but in all T2-hypointense GM regions (globus pallidus, substantia nigra, red nucleus, dentate nucleus). The striking pronunciation of age-related increase in T2-hypointensity within the dentate nucleus is well explained by the different kinetics of age-related iron accumulation across different GM areas given that the age of our subjects ranged between 20 and 58. In the basal ganglia and thalamus, a significant increase in iron content was observed only after the age of forty [38], while a considerable increase in iron content beginning as early as the age of 20 years was observed in the dentate nucleus [36]. Of note, our approach identified age-related T2-hypointensity better than both the conventional frequentist approach as implemented in SPM8 whereas the Bayesian approach did not work properly. The frequentist approach did not yield meaningful results at the pre-defined statistical threshold of FDR 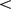
0.05. Age-related changes of T2-hypointensity could only be visualized at unacceptably liberal statistical thresholds up to an uncorrected 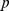-value of 0.05. Even though our results showed that the simple GMRF prior clearly improves the estimation of regression coefficients, we note that the specified prior for the regression coefficients can have troublesome features [39] and alternative strategies may be more effective. Moreover, the proposed voxel-wise regression model can be extended in various ways. For example, better edge preserving properties may be achieved by introducing spatially adaptive interaction weights between adjacent voxels [8]. Further, spatial and non-spatial prior information can be combined in order to separate the control over the variance and the effect of neighboring voxels [40]. The use of Diffusion-based spatial priors [41] may also improve the estimation. With regard to possible non-linear relations, more realistic modeling can be achieved by P-Splines [42] or alternative distributional assumptions for the response variable.

In summary, we have demonstrate that fully Bayesian inference can successfully be applied for preprocessing and statistical analysis of structural MRI data.
